# Caspase Inhibition with XIAP as an Adjunct to AAV Vector Gene-Replacement Therapy: Improving Efficacy and Prolonging the Treatment Window

**DOI:** 10.1371/journal.pone.0037197

**Published:** 2012-05-16

**Authors:** Jingyu Yao, Lin Jia, Naheed Khan, Qiong-Duan Zheng, Ashley Moncrief, William W. Hauswirth, Debra A. Thompson, David N. Zacks

**Affiliations:** 1 Department of Ophthalmology and Visual Sciences, University of Michigan Medical School, Ann Arbor, Michigan, United States of America; 2 Department of Biological Chemistry, University of Michigan Medical School, Ann Arbor, Michigan, United States of America; 3 Department of Ophthalmology, University of Florida College of Medicine, Gainesville, Florida, United States of America; Massachusetts Eye & Ear Infirmary, Harvard Medical School, United States of America

## Abstract

**Purpose:**

AAV-mediated gene therapy in the *rd10* mouse, with retinal degeneration caused by mutation in the rod cyclic guanosine monophosphate phosphodiesterase β-subunit (PDEβ) gene, produces significant, but transient, rescue of photoreceptor structure and function. This study evaluates the ability of AAV-mediated delivery of X-linked inhibitor of apoptosis (XIAP) to enhance and prolong the efficacy of PDEβ gene-replacement therapy.

**Methods:**

*Rd10* mice were bred and housed in darkness. Two groups of animals were generated: Group 1 received sub-retinal AAV5-XIAP or AAV5-GFP at postnatal age (P) 4 or 21 days; Group 2 received sub-retinal AAV5-XIAP plus AAV5- PDEβ, AAV5-GFP plus AAV5- PDEβ, or AAV- PDEβ alone at age P4 or P21. Animals were maintained for an additional 4 weeks in darkness before being moved to a cyclic-light environment. A subset of animals from Group 1 received a second sub-retinal injection of AAV8-733-PDEβ two weeks after being moved to the light. Histology, immunohistochemistry, Western blots, and electroretinograms were performed at different times after moving to the light.

**Results:**

Injection of AAV5-XIAP alone at P4 and 21 resulted in significant slowing of light-induced retinal degeneration, as measured by outer nuclear thickness and cell counts, but did not result in improved outer segment structure and rhodopsin localization. In contrast, co-injection of AAV5-XIAP and AAV5-PDEβ resulted in increased levels of rescue and decreased rates of retinal degeneration compared to treatment with AAV5-PDEβ alone. Mice treated with AAV5-XIAP at P4, but not P21, remained responsive to subsequent rescue by AAV8-733-PDEβ when injected two weeks after moving to a light-cycling environment.

**Conclusions:**

Adjunctive treatment with the anti-apoptotic gene XIAP confers additive protective effect to gene-replacement therapy with AAV5-PDEβ in the *rd10* mouse. In addition, AAV5-XIAP, when given early, can increase the age at which gene-replacement therapy remains effective, thus effectively prolonging the window of opportunity for therapeutic intervention.

## Introduction

Retinitis pigmentosa (RP) encompasses a heterogeneous group of inherited retinal dystrophies characterized by photoreceptor dysfunction and eventual photoreceptor death [1]. Strategies aimed at preventing the cell death with anti-apoptotic agents have shown some success in animal models, but have not yet been translated into practical treatments for humans [2]. Newly introduced experimental therapies such as treatment with neurotrophic factors [3], [4] and targeted gene–replacement offer hope for improved outcomes [5], [6], [7]. The latter approach is particularly appealing in that a normal copy of the gene is introduced that corrects a functional deficit caused by a loss-of-function mutation. However, development of targeted gene-replacement therapy is a daunting task, given that RP is known to be caused by mutations in over 100 genes [8]. Thus, the clinical reality is that patients will continue to experience disease progression until a corresponding gene therapy vector can be developed. Thus, there remains an urgent need for a complementary strategy to prevent photoreceptor cell death until a definitive treatment is developed. In essence, we need a way to prolong the window-of-opportunity-for treating the disease.

The X-linked inhibitor of apoptosis (XIAP) is a key member of the gene family of inhibitors of apoptosis. XIAP is involved in binding to and suppressing the activity of caspases 3, 7, and 9 [9]. Previous studies have shown that adeno-associated viral (AAV) constructs encoding XIAP exert protective effects in various models of retinal injury and disease including retinal ischemia [10], ganglion cell death induced by axotomy [11], [12] or increased intraocular pressure [13], chemotoxic insult 14,15, retinal detachment [16], retinal cell transplantation [17], and in genetic models of retinitis pigmentosa [18]. What is not yet known is whether XIAP-protected cells can be subsequently rescued by transduction with a vector carrying a normal copy of the disease-causing mutant gene.

The retinal degeneration 10 (*rd10*) mouse is a model of autosomal-recessive RP caused by a point mutation of the *Pde6b* gene[19]. Death of photoreceptors in the *rd10* mouse begins soon after birth, with most photoreceptor cells lost by 5 weeks of age [20], [21]. Dark-rearing delays the onset of degeneration by as much as 4 weeks [19], however, rapid onset of photoreceptor degeneration occurs as soon as the animals are moved to a normal 12 h/12 h cycling light environment. The *rd10* phenotype can be rescued with sub-retinal injection of AAV- PDEβ [22], [23], and is especially effective when dark-rearing is used to achieve onset of gene transduction and maximal protein synthesis prior to onset of the degeneration.

The goal of the present study is to determine if anti-apoptotic therapy with AAV5-XIAP can prolong the window-of-opportunity for treatment of a hereditary retinal degeneration. Our results show that transduction of photoreceptors in the *rd10* mouse with AAV5-XIAP confers structural protection of the outer nuclear layer. In addition, AAV5-XIAP treated photoreceptors can be transduced subsequently with either AAV5- PDEβ or AAV8-733-PDEβ, in effect showing that AAV5-XIAP can prolong the window of opportunity for gene replacement therapy. We conclude that adjunctive treatment with the anti-apoptotic gene XIAP has an additive effect on gene-replacement therapy with AAV- PDEβ alone.

## Methods

### Animals

*Rd10* mice, and C57BL/6 mice that served as wild-type controls, were obtained from The Jackson Lab (Jackson Laboratories, Bar Harbor, ME, USA). Mice were bred and housed in the University of Michigan, Kellogg Eye Center animal facility. All animal experiments were conducted in accordance with the ARVO statement for the Use of Animals in Ophthalmic and Vision Research and approved by the University Committee on the Use and Care of Animals of the University of Michigan.

### Vectors and Experimental Animal Groups

AAV5-XIAP (AAV5-CBA-XIAP-wPRE, HA tagged XIAP), AAV5-GFP (AAV5-CBA-GFP), AAV5- PDEβ (AAV5-smCBA-PDEβ) and AAV8-733- PDEβ (AAV8-smCBA- PDEβ in which a Y to F capsid amino-acid change was made at position 733) were produced and purified as previously described [15]–[17], [22], [23]. Late-term (around embryonic day 14) pregnant *rd10* females were moved from a cyclic-light environment (12-hour light/12-hour dark) into a continuously dark room. Two groups of animals were generated: Group 1 received sub-retinal injection of AAV5-XIAP or AAV5-GFP at postnatal age (P) 4 or 21 days; Group 2 received sub-retinal injection of AAV5-XIAP plus AAV5-PDEβ, AAV5-GFP plus AAV5-PDEβ, or AAV5-PDEβ alone at age P4 or P21. After injection, the animals were maintained for 4 weeks in the dark before they were moved to a normal 12 h/12 h light/dark cycling environment. A subset of animals in Group 1 received a second sub-retinal injection of AAV8-733-PDEβ at two weeks after being moved to the light.

### Sub-retinal Injection

Sub-retinal injections were performed as previously described [17]. Age P4 pups were anesthetized by hypothermia. The eye lids were cleaned with 70% ethanol using a cotton-tipped applicator and the interface of the upper and lower lids were cut with a sterile scalpel. A sclerotomy was made in the superior sclera just posterior to the cornea using the tip of a 30-gauge needle. A tapered pulled glass pipette was inserted into the sclerotomy across the vitreous to the opposite wall of the eye until resistance was encountered. The pipette was slightly retracted from that point to position it in the sub-retinal space. The pipette was connected to a nanoinjector (Nanoinjector II, Drummond Scientific Company), which allowed for a slow injection of the vector (1 ul containing 1×10^10^ vector DNA containing AAV particles) into the sub-retinal space.

Mice age P21 or older were anesthetized by intra-peritoneal injection of an anesthetic cocktail that consisted of ketamine (100 mg/ml, Fort Dodge Animal Health, USA), xylazine (LLOYD, Inc., IA) and sterile PBS in the ratio of 8∶8∶34. The dose of cocktail injected was 0.1 ml per 20 g body weight. The sub-retinal injection was performed as described above with direct visualization through a dissecting microscope. The other eye remained uninjected. After all injections, 1% atropine eye drops and chloramphenicol ophthalmic ointment were given. Only animals with minimal surgical complications were retained for further evaluation. At least four animals were included for each time point of each experimental group.

### Immunocytochemistry

Mice were sacrificed and the eyes enucleated before and at 1, 2, 3 and 6 weeks after moving to the light, as follows: Animals were deeply anesthetized and then perfused transcardially with 4% paraformaldehyde in PBS. Eyes were collected and immersion fixed with 4% paraformaldehyde at room temperature for 30 min. The cornea and lens were removed and the eyecup rinsed three times in PBS, then transferred to 10% and then 20% sucrose in PBS for 2 h each, before embedding in OCT (Tissue Tek) mixed in a ratio of 1∶1 with 20% sucrose. A cryostat was used to obtain 10 µm thick serial retina/RPE/choroid sections.

Sections were washed in PBS, blocked with 10% goat serum and 0.1% Triton-X 100 for 1 hr, and incubated with primary antibodies diluted with 10% goat serum overnight at 4°C. The primary antibodies and working dilutions were as follows: anti–PDEβ (1∶400, ABR-Affinity BioReagents, Golden, CO), mouse monoclonal rho1D2 (1∶1000, a kind gift from Dr. Robert Molday), and anti-hemagglutinin (HA) (1∶100, Roche Applied Science, Laval, QC, Canada) that is used as an expression tag for the AAV5-XIAP vector. After three 10-min rinses with 0.1% Triton-X 100, sections were incubated for 90 min at room temperature with secondary antibodies diluted 1∶1000 in PBS containing 0.1% Triton X-100. The secondary antibodies were: goat anti-mouse IgG, Alexa Fluor 647, (Invitrogen, USA) and goat anti-rabbit IgG (H+L) Alexa Fluor 546 (Invitrogen, USA), goat anti-rat Alexa Fluor 488 (Invitrogen, USA). Sections were counter-stained with ProLong Gold with DAPI (Invitrogen, USA) to reveal cell nuclei.

### Microscopy and Outer Nuclear Layer Thickness Comparison

Retinal images were obtained using a confocal microscope (Leica SP5, Leica Corp., Germany). Three representative sections encompassing the optic nerve were used from each eye. The total thickness of the outer nuclear layer (ONL) and retina (from the outer edge of the ONL to the inner limiting membrane) was measured in three places in each of three non-overlapping high-power fields (40×) images at the comparable area per section and averaged for each eye. Photoreceptor inner and outer segments were not included in the total retinal thickness measurement. The thickness of the ONL was normalized to total retinal thickness, thus minimizing possible variability in measurement between samples that could be caused by differences in angle of sectioning. For each experimental group, measurements were made on three sections of 3 to 5 eyes; each eye was from a different animal.

### Western Blot Analysis

Retinas were dissected from treated and untreated *rd10* eyes at various time points. Age-matched normal C57BL/6J eyes were used as controls. Samples were homogenized in hypotonic buffer, and the proteins separated by 10% SDS-PAGE and blotted onto nitrocellulose. The membranes were incubated overnight with: anti-PDEβ (1∶500, ABR-Affinity BioReagents, Golden, CO), anti-HA(1∶200, Roche Applied Science, Laval, QC, Canada), anti-GC1(1∶200, Santa Cruz Biotechnology, Inc.), anti-rho (1∶400) (gift from Dr. Barry Knox), anti-transducin (1∶1000), or anti-PDEa (1∶300) (gifts from Dr. Jose Bubis). The blots were developed with alkaline phosphatase development buffer supplemented with 1% v/v BCIP and 1% v/v NBT. Treated, untreated *rd10* and normal C57BL/6J samples were compared on the same blot with GAPDH as an internal loading control.

### Caspase 3 Activity Assays

One cohort of mice that received sub-retinal AAV5-XIAP injection at P4 was used for caspase 3 activity assays at 24 h after moving to the light. Total retinal protein from AAV5-XIAP treated or untreated eyes was extracted as previously described.^11^ Caspase 3 activity was measured with a colorimetric assay kit (Chemicon International, Billerica, MA), in accordance with the manufacturer's instructions. This assay is based on cleavage of the pNA-DEVD substrate by activated caspase 3. For each group, the data represented the average caspase 3 activity levels of protein from 18 eyes.

### Electroretinography

Full-field electroretinograms (ERGs) were recorded at various time points (before and at 1, 2, 3 and 6 weeks after moving to the light). Mice were dark-adapted overnight and prepared under dim-red illumination. Animals were anesthetized with an intra-peritoneal loading dose of ketamine and xylazine. Pupils were dilated with topical 1% atropine and 0.5% tropicamide. Body temperature was maintained at 37°C with a heating pad during the course of the experiment. Corneal ERGs were recorded from both eyes using gold wire loops with 0.5% tetracaine eye drops for topical anesthesia and a drop of 2% methylcellulose for corneal hydration. A gold wire loop placed in the mouth was used as reference, and ground electrode was on the tail. ERGs were recorded with a Ganzfeld configuration using the Espion e^2^ recording system (Diagnosys, Lowell, MA) to brief xenon white flashes. Responses were amplified at 1,000× gain at 1.25 to 1000 Hz, and digitized at a rate of 2000 Hz. A notch filter was used to remove 60 Hz line noise. Dark-adapted ERGs were recorded at a dim flash of −2.31 log cd-s/m^2^/flash with an inter-stimulus interval (ISI) of 4 seconds, and 15 sweeps were averaged. Three sets of recordings were obtained to ensure reproducibility. A bright flash at 1.09 cd-s/m^2^/flash, with a maximum of 10 sweeps/average and ISI of 30 seconds were recorded to elicit a maximum response. Light-adapted responses were recorded after 10 min of adaptation to a white 32 cd/m^2^ rod-suppressing background for a flash intensity of 1.09 log cd-s/m^2^/flash and three sets of 20 sweeps averaged. The amplitudes of scotopic and photopic B-waves of injected eyes were all normalized by dividing by the scotopic and photopic B-waves of untreated eye of the same animal before moving to the cycling light-dark environment to minimize variability among individuals.

### Statistical Analysis

Comparison of normalized ONL thickness (thickness of ONL/thickness of retina) and normalized ERG B-wave amplitudes at each time point for different vector treated groups were performed using analysis of variance (ANOVA) to provide pairwise comparisons with Sheffe's adjustment for multiple comparisons. Non-parametric Kruskal-Wallis (K-W) tests were performed for overall differences in normalized ONL thickness among the groups at each time. Significance was defined as a p-value of less than 0.05. Comparison of caspase 3 activity was performed using two-tailed Student's *t*-tests without assuming equal variance. Differences were considered significant at *P*<0.05.

## Results

### AAV5-XIAP inhibits caspase activity and increases photoreceptor survival in *rd10* mice

**Figure 1:**
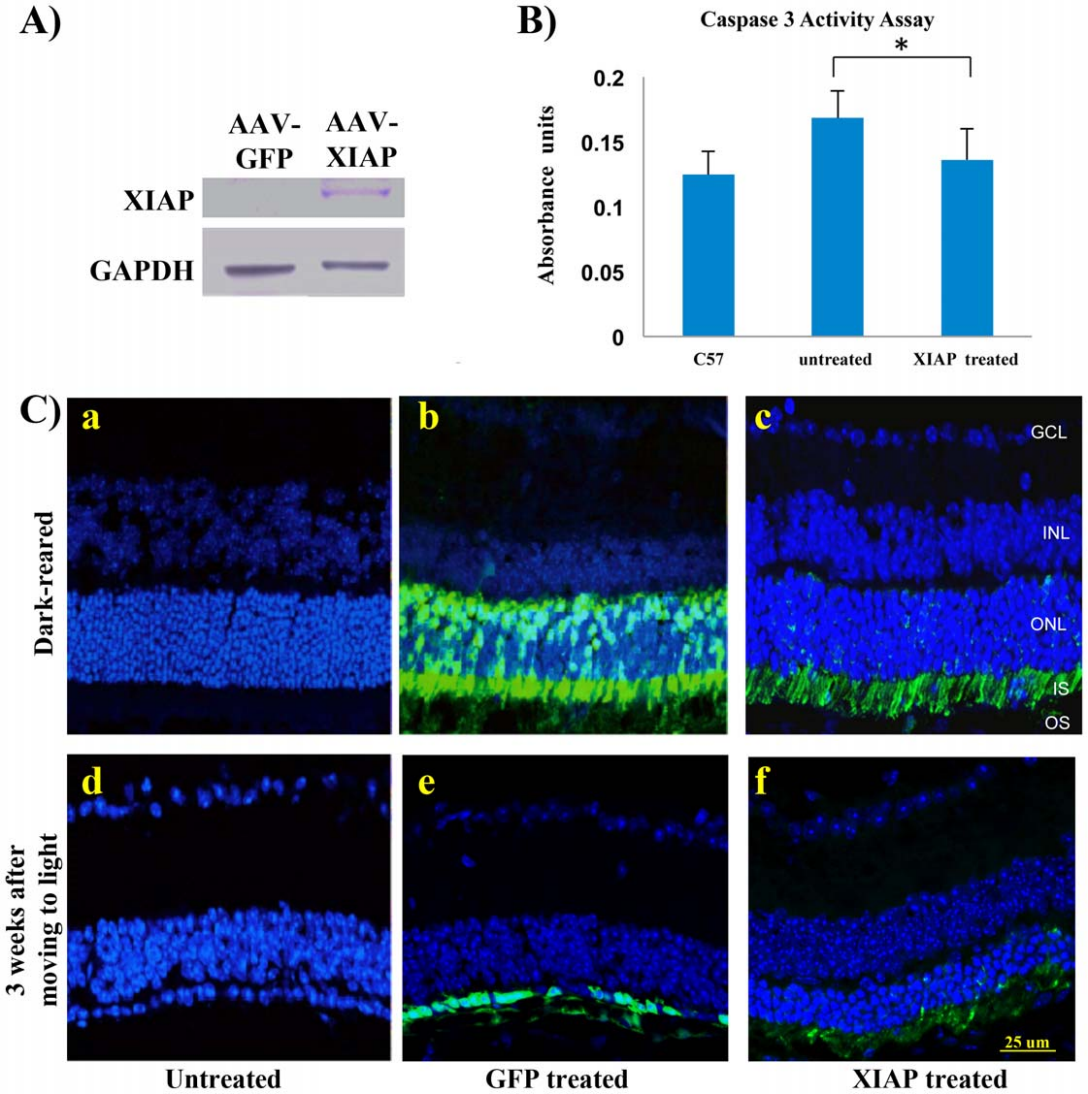
Effects of subretinal injection of AAV5-XIAP, or a control AAV5-GFP construct, on *rd10* mice treated at P4 and dark-reared for 4 weeks post treatment before being moved to the light environment. **A.** Western-blot analysis shows expression of recombinant XIAP. **B.** Caspase 3 activity assayed 24 hours after transfer from the dark-rearing to the light environment shows AAV5-XIAP reduced caspase 3 activity levels in untreated *rd10* retinas to levels found in control C57 mice. **C.** Retinal histology at 3 weeks after moving to the light environment shows extensive degeneration of the ONL in untreated (a,d) and AAV5-GFP treated retinas (b,e; green shows localization of GFP), and significant preservation of ONL thickness and cell counts in the AAV5-XIAP treated retinas (c,f; green showing localization of XIAP by staining of HA-tag).

To test the ability of AAV5-XIAP to transduce the photoreceptor cells and inhibit the downstream activation of apoptosis, *rd10* mice received a sub-retinal injection with AAV5-XIAP at P4 and were kept in the dark for 4 weeks to allow for gene transduction and maximal protein synthesis. Injection with AAV5-GFP served as a control. Western blotting confirmed that the XIAP protein was overexpressed in the retinas of AAV5-XIAP injected eyes and undetectable in the contralateral untreated eyes (Figure 1A). Assays of caspase 3 activity 24 h after moving the animals to the light showed significantly increased levels at 24 h in *rd10* mice as compared to wild type, and these increases were significantly reduced by AAV5-XIAP treatment (Figure 1B, *p* = 0.04). Immunohistochemistry showed that the XIAP expression was confined to the photoreceptors and localized primarily to their inner segments (Figure 1C-c), consistent with prior reports of XIAP localization to this photoreceptor compartment [16], [17]. GFP expression was present in the photoreceptor inner segments and throughout the cell bodies (Figure 1C-b).

Retinas from *rd10* mice treated with AAV5-XIAP at P4 showed significant structural preservation as compared to those treated with AAV5-GFP. Treated and untreated animals reared in darkness showed nearly normal retinal structure (Figure 1C a–c). However, after moving the animals to the light environment, both the AAV5-GFP injected eyes and untreated eyes showed severe degeneration (Figure 1C d–e). In contrast, retinas from mice treated with AAV5-XIAP were protected, with significantly greater photoreceptor preservation as compared to control or GFP-treated animals (Figure 1C-e)

**Figure 2:**
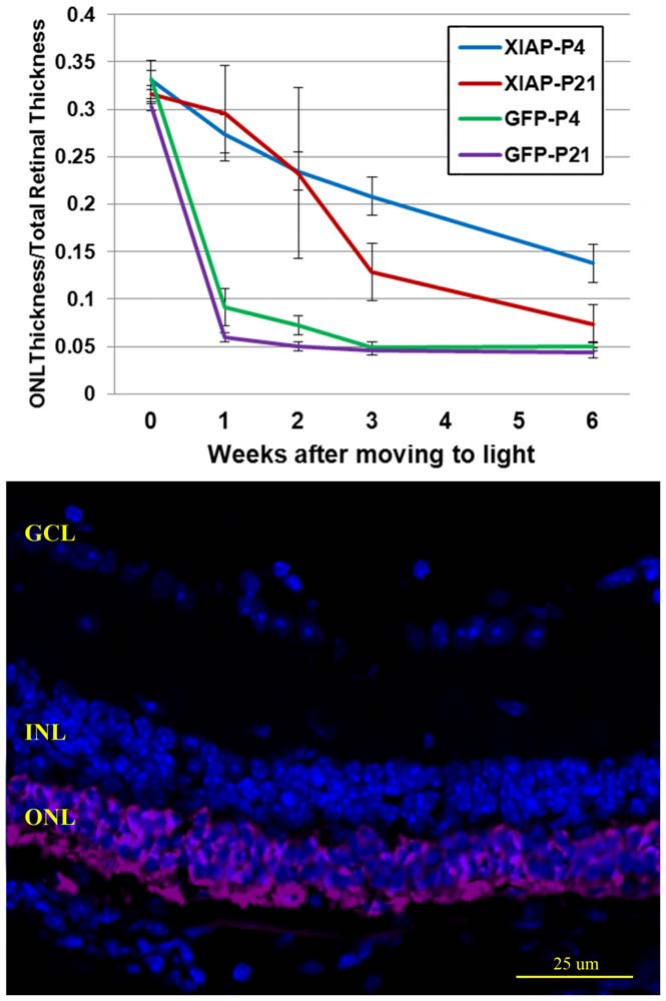
Effects of sub-retinal injection of AAV5-XIAP on photoreceptor cell survival and rhodopsin expression in *rd10* mice. **Top Panel.** The ratio of ONL thickness to total retinal thickness in *rd10* retinas transfected with AAV5-GFP or AAV5-XIAP. Animals received the sub-retinal injection at either P4 or P21 and then were maintained in the dark-rearing environment for 4 weeks before being moved to the light environment (time = 0). **Bottom Panel.** *rd10* retina after AAV5-XIAP treatment shows rhodopsin mislocalization throughout the ONL and shortened outer segments. (Rhodopsin, purple; DAPI, blue).

AAV5-XIAP provided greater photoreceptor protection when animals were treated at P4 as compared to P21 (Figure 2, Top Panel). Regardless of the age at treatment, the rate of decrease of ONL thickness was significantly slower after AAV5-XIAP transduction than after AAV5-GFP transduction (p<0.001), in which only one layer of photoreceptor nuclei remained after one week in the light environment. However, the morphology of the XIAP-treated retinas was not normal, with photoreceptors displaying a marked absence of outer segments. Western blots of retinal protein extracts showed rhodopsin was present (data not shown), but immunohistochemical analysis revealed only a diffuse distribution of rhodopsin staining throughout the ONL and inner segments (Figure 2, Bottom Panel).

**Figure 3:**
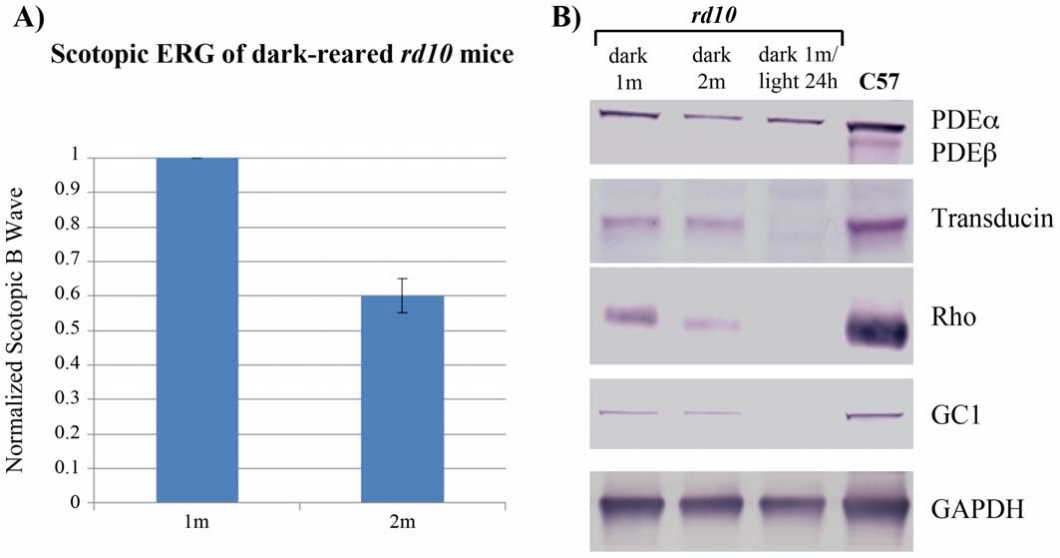
Retinal function and protein expression in 1 and 2 month-old dark-reared *rd10* mice. **A.** Normalized scotopic ERG B-wave responses showing age-dependent loss-of-function under conditions where ONL cell counts were preserved. **B.** Western blots of PDEα, transducin, rhodopsin (Rho) and guanylate cyclase 1 (GC1) expression showing significant age-dependent losses occurring in the dark, and catastrophic losses occurring 24 hours after moving to the light environment.

The slower rate of degeneration found in eyes treated with AAV5-XIAP at P4 as compared to P21 could reflect the fact that the animals treated at P21 were 17 days older than the P4-treated animals when moved to the light. This is consistent with our analysis of visual transduction protein levels and scotopic ERGs, which were significantly lower in animals dark-reared for two months compared to one month (Figure 3A, B). The measurable ERG response of dark-reared *rd10* mice, despite the absence of a functional PDEβ subunit, can likely be attributed this to the presence of a normal PDEa subunit, and its ability to act as a substitute for the missing PDEβ subunit [24]. After moving the *rd10* mice to the light environment, there was a complete loss of ERG responses from both the untreated and GFP-treated animals within several days. Though PDEa was still present, movement to light resulted in a rapid loss of other visual transduction proteins such as rhodopsin, transducin and guanylate cyclase 1 (Figure 3A). Despite the slower rate of cell death in the AAV5-XIAP treated animals, ERG responses were also lost, consistent with the observed loss of photoreceptor outer segments in these animals (data not shown).

### AAV5-XIAP enhances AAV5- PDEβ rescue of photoreceptor structure and function

**Figure 4:**
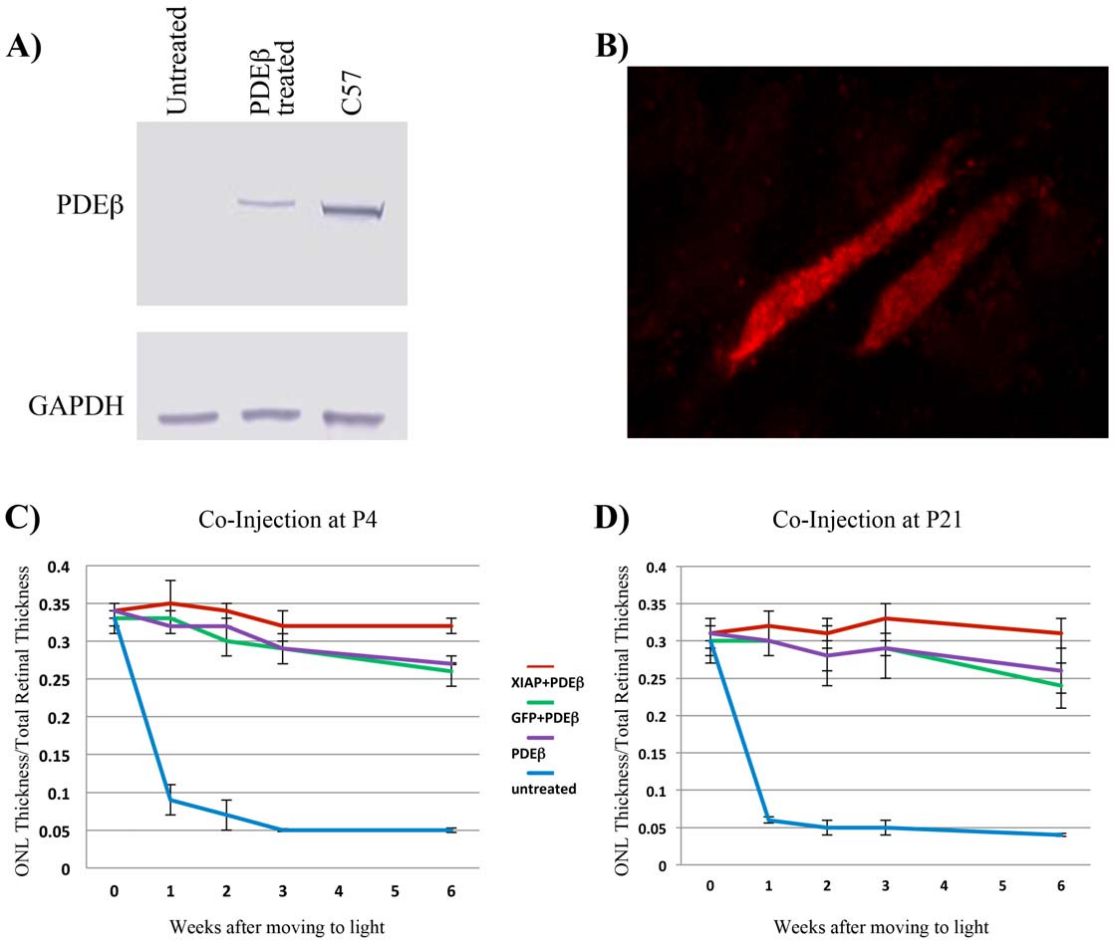
Co-transduction of *rd10* retinas with AAV5-PDEβ and AAV5-XIAP at P4 and P21. **A.** Western blot of PDEβ expression in AAV5-PDEβ transduced *rd10* retinas compared to wild type (C57) retinas. **B.** Confocal imaging of isolated rod outer segments from AAV5-PDEβ transduced *rd10* retinas showing expression of PDEβ (red). **C–D.** Co-treatment of *rd10* retinas with AAV5-XIAP and AAV5-PDEβ showing improved survival of photoreceptor cells compared to retinas treated with AAV5-PDEβ alone or in combination with AAV5-GFP. **C.** Co-injection at PN4. **D.** Co-injection at PN21.

**Figure 5:**
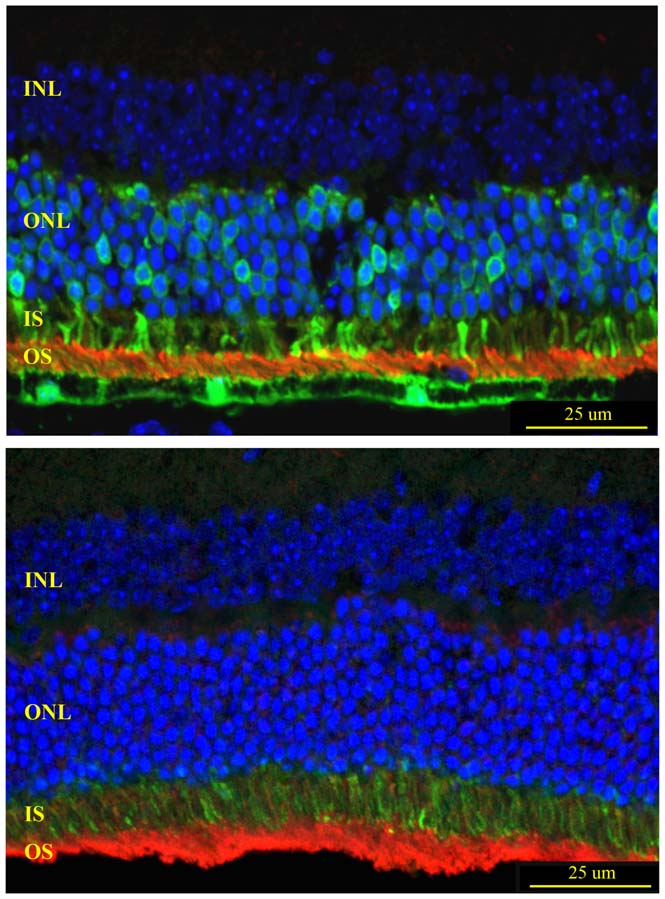
Retinal histology of AAV5-PDEβ transduced *rd10*retinas. **Top Panel.** Sub-retinal injection of AAV5-PDEβ co-treated with AAV5-GFP resulted in significant preservation of outer nuclear layer and inner and outer segment morphology. (GFP, green; PDEβ, red). **Bottom Panel.** Co-injection with AAV5-XIAP and AAV5-PDEβ shows improved preservation of retinal structures. XIAP (green – via staining of HA-tag) and PDEβ (red) localized to inner and outer segments, respectively.

Consistent with the results of previous studies [22], [23], sub-retinal injection of AAV5-PDEβ resulted in the expression of PDEβ within the photoreceptors of *rd10* retinas (Figure 4A,B) and the significant preservation of photoreceptor cell numbers (Figure 4C,D). In contrast to treatment with AAV-XIAP, AAV-PDEβ treatment resulted in improved photoreceptor morphology, including the presence of both inner and outer segments (Figure 5, Top Panel). Furthermore, retinas treated with AAV-PDEβ exhibited normal localization of rhodopsin to the outer segments (not shown). However, AAV-PDEβ treated retinas still underwent slow but significant photoreceptor degeneration. After 6 weeks in the light environment, ONL thickness decreased by approximately 20 percent in both the P4 and P21 treated animals (Figure 4C,D).

Co-treatment with AAV5-XIAP significantly reduced the degeneration of the AAV5- PDEβ treated retinas in both the P4 and P21 cohorts (Figure 4C,D). At six weeks after moving to the light, the ONL thickness of retinas co-treated with AAV5-XIAP remained stable, with no detectable diminution as compared to baseline (prior to moving to the light). Co-treatment of AAV5-PDEβ with the control vector AAV5-GFP showed a similar pattern of photoreceptor degeneration after transfer to the light as was observed in retinas treated with AAV5-PDEβ alone. Immunohistochemical analysis of co-treated retinas showed co-localization of PDEβ with XIAP or GFP, verifying the co-transduction of cells with each of the two separate vectors (Figure 5, Bottom Panel).

**Figure 6:**
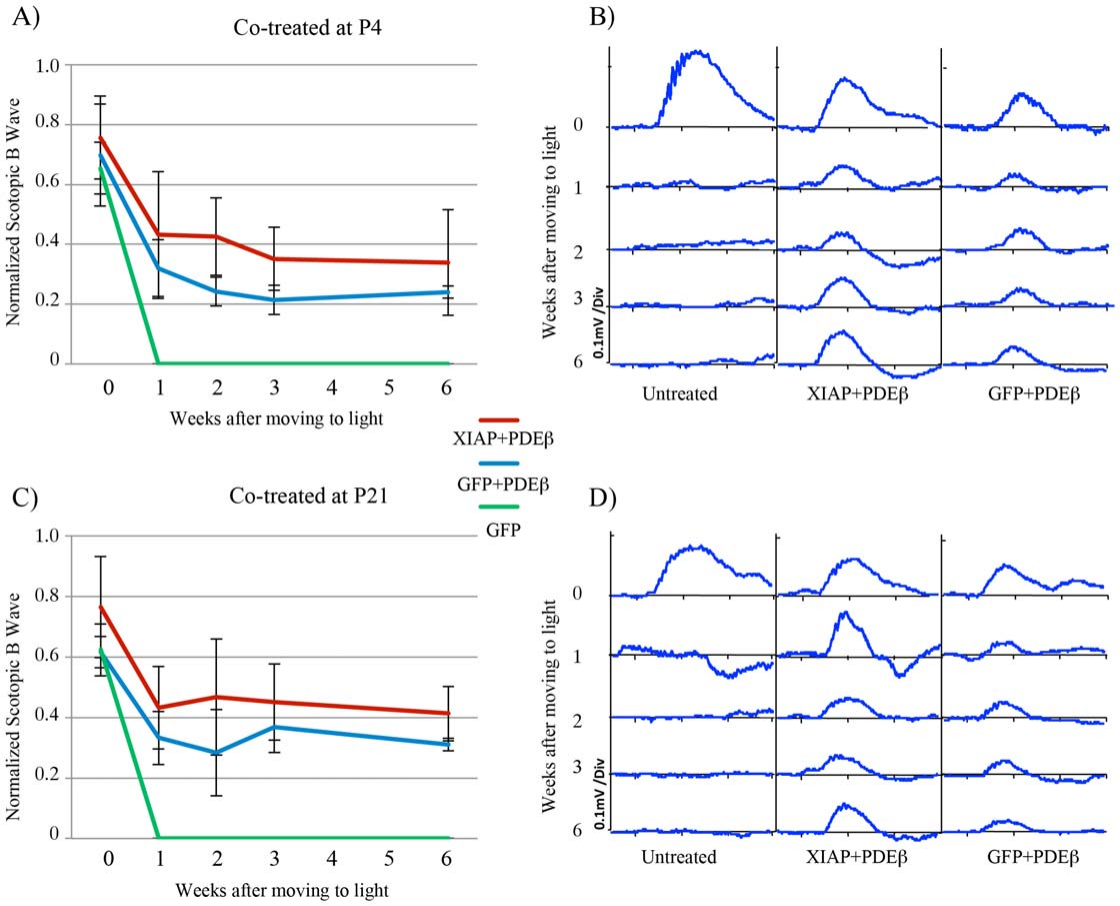
ERG analysis of retinal function in *rd10* mice treated by co-injection of AAV5-XIAP and AAV5-PDEβ. **A, C.** Scotopic B wave responses of treated mice at 0, 1, 2, 3 and 6 weeks after being moved to the light-environment were normalized to the scotopic B wave of untreated retinas at time 0. Responses in mice treated with AAV5-XIAP plus AAV5-PDEβ were greater and maintained longer that those of mice treated with AAV5-GFP plus AAV5-PDEβ. The absence of rescue by AAV5-GFP alone excludes the possibility that rescue results from the surgical procedure alone or exposure to intact vector. Similar outcomes were obtained in mice treated at P4 and P21. **B, D.** Representative ERG tracings of *rd10* mice treated with AAV5-XIAP plus AAV5-PDEβ, AAV5-GFP plus AAV5-PDEβ, or untreated, at 0, 1, 2, 3 and 6 weeks after being moved to the light environment. The response of untreated retinas at t = 0 was larger than the response of treated retinas, presumably because of some deleterious effects caused by the transient retinal detachment that necessarily accompanies sub-retinal vector injection. Untreated retinas, however, quickly lose all ERG responses, with no detectable B wave left by 1 week after moving into the light.

Treatment with AAV5-PDEβ preserved retinal function, and this was enhanced by co-treatment with AAV5-XIAP. Scotopic and photopic ERGs were measured prior to moving animals from dark-rearing to the light environment (time 0), and then at 1, 2, 3 and 6 weeks after moving to the light (Figure 6A,C). The amplitudes of scotopic and photopic B-waves of injected eyes were all normalized by dividing by the scotopic or photopic B-waves amplitudes of untreated eye of the same animal at time zero, in order to minimize the variability among animals. Untreated eyes showed higher ERG responses compared to treated eyes at time zero, suggesting that perhaps the surgical procedure or the AAV-vector has a deleterious effect on the retina (Figure 6B,D). After transferring to the light, however, the ERGs of untreated eye were quickly extinguished, consistent with the rapid photoreceptor degeneration (Figure 6B,D). Treatment with AAV5-PDEβ at either P4 or P21 preserved retinal function. Consistent with the improved survival of photoreceptor cells in AAV5-PDEβ treated retinas with adjunctive AAV5-XIAP, these co-treated retinas had improved ERG function as compared to retinas co-treated with AAV5-GFP (Figure 6A,C). There was no significant difference in the ERG responses of P4 versus P21 treated retinas.

### Sequential treatment of AAV5-XIAP followed by AAV8-733-PDEβ

*Rd10* mice in this treatment group received an initial sub-retinal injection of AAV5-XIAP at age P4 or 21, were maintained for 4 weeks in the dark, and then moved to the light. After two weeks in the light, the animals received a sub-retinal injection of AAV8-733-PDEβ into the same eye. The AAV8-733 serotype was used for the second injection because of its more rapid and efficient expression of transgene as compared to the AAV5 serotype [23]. Control animals received AAV8-733-PDEβ at 2 weeks after transfer to light, but without initial XIAP treatment. Histological, immunohistochemical, and ERG analysis were performed at 4 weeks after the second sub-retinal injection (6 weeks after moving to the light). The transduction efficiency of the sequential injection of AAV vectors into the same eye was evaluated relative to sub-retinal injection of AAV5-GFP in C57 mice followed by a second injection of AAV8-733-CHERRY at matched experimental time points. There was over 75% overlap of GFP and CHERRY staining in photoreceptor cells, indicating that vectors of different serotype, and which express different transgenes, can be delivered sequentially into the same cell effectively (data not shown).

**Figure 7:**
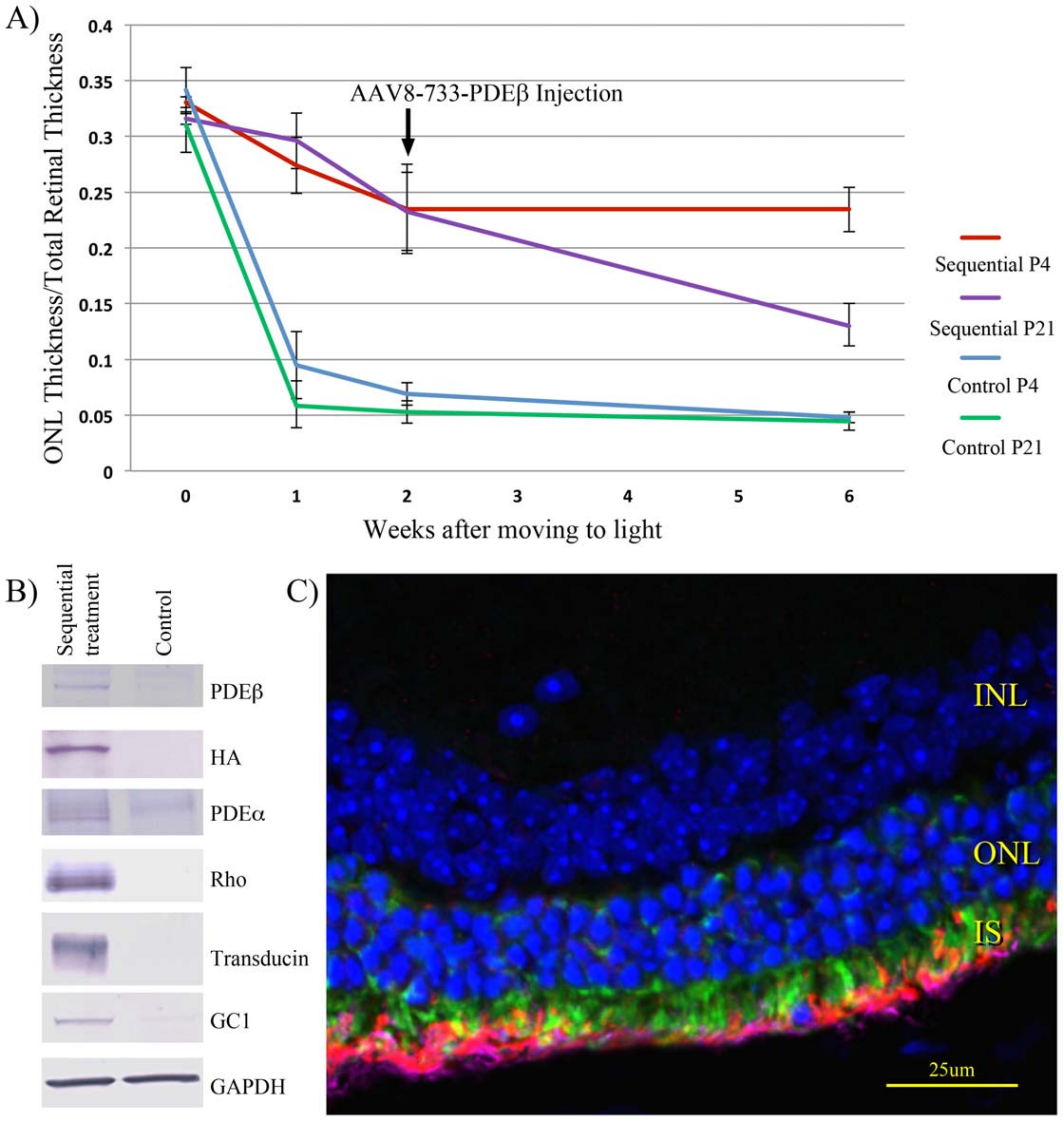
Sequential treatment of *rd10* mice with AAV5-XIAP and AAV8-733-PDEβ. Dark-reared *rd10* mice were treated with AAV5-XIAP by sub-retinal injection at age P4 or P21, and then maintained in darkness for 4 weeks before being moved to the light environment. After 2 weeks in the light, the mice were treated with AAV8-733-PDEβ by sub-retinal injection, and then evaluated at the times indicated. Control eyes received only the AAV8-733-PDEβ treatment two weeks after being moved to the light environment. **A.** Thickness measurements show slowing of ONL cell losses by treatment of *rd10* mice with AAV5-XIAP alone. In animals treated with AAV5-XIAP at age P4, but not at P21, further losses of ONL thickness were halted by the subsequent administration of AAV8-733-PDEβ. **B.** Western blots of retinas sequentially injected with AAV5-XIAP and AAV8-733-PDEβ, and examined 4 weeks later, show expression of recombinant XIAP and PDEβ in transduced retinas, and preservation of the expression of endogenous PDEa, rhodopsin (Rho), transducin and guanylate cyclase 1 (GC1). **C.** Histology of retinas sequentially injected with AAV5-XIAP and AAV8-733-PDEβ, and examined 4 weeks later, shows localization of XIAP (green – via staining of HA-tag) to inner segments and PDEβ (red) and rhodopsin (purple) to outer segments. Outer segments appear to be shortened, and there is mislocalization of PDEβ and rhodopsin to the inner segments.

As described above, retinas treated with only AAV5-XIAP exhibited a slower rate of ONL degeneration upon moving to the light, as compared to control or untreated eyes. Subsequent injection of AAV8-733-PDEβ two weeks later prevented further loss of photoreceptors in retinas that received the initial AAV-XIAP treatment at P4 (Figure 7A). However, sequentially injected animals treated with XIAP at P21 showed continued degeneration. No structural preservation was seen in control retinas. Western analysis of the retinas from animals injected with AAV-XIAP at P4 showed that the levels of visual transduction proteins were stabilized by the subsequent transduction with AAV8-733-PDEβ (Figure 7B). Histologic analysis of the P4-injected cohort at the six week time point (four weeks after the second sub-retinal injection) showed preserved photoreceptor cells but marked disruption of the outer segment morphology (Figure 7C). XIAP was expressed in the inner segments and PDEβ was found in the distorted outer segments. Rhodopsin staining could also be detected. However, no ERG activity was detected due, perhaps at least in part, to the disorganization of photoreceptor structure potentially caused by multiple sequential injections into the sub-retinal space.

## Discussion

Previous attempts to improve outcomes in hereditary retinal degenerations using anti-apoptotic or neurotrophic therapies have focused on evaluating their ability to prevent or slow rates of photoreceptor cell death [2], [25]. While important advances have been made, this strategy has not yet proven itself to be a stand-alone therapy. The reasons for this may vary, depending on the disease-causing mutation, but could include the toxic build-up of proteins or metabolic by-products, or the activation of secondary death pathways which ultimately result in cell death [25]. Most importantly, anti-apoptotic therapy cannot replace activity lost due to loss-of-function mutations, so deficits will still exist even if the cells survive. On the other hand, vector-mediated gene-replacement is a relatively new therapeutic technique, and its strengths and limitations in reconstituting function and promoting long-term photoreceptor survival are not yet well established in a broad range of different genetic models. The current study explores the effect of combining these two approaches, and finds that adjunctive use of XIAP can both extend the window-of-opportunity and enhance the effects of gene-replacement therapy.

On its own, we find that subretinal injection of AAV5-XIAP results in a delay of the catastrophic light-dependent photoreceptor-cell death occurring in *rd10* mice, but it cannot preserve ERG responses once the mice are moved from the dark to the light. The presence of ERG responses in dark-reared *rd10* mice, in which the disease-causing mutation results in PDEβ loss-of-function, likely reflects the ability of PDEa to act as a surrogate in generating the functional enzyme [24]. Even though photoreceptor nuclei and inner segments are initially well preserved, the inability of AAV5-XIAP to prevent loss of the ERG is likely due to the fact that the shift to the light cycling environment results in rhodopsin mislocalization and loss/shortening of outer segments. A second explanation, and one that is not mutually exclusive, is that the light-induced loss of key photoreceptor proteins, including transducin and guanylate cyclase, precludes the generation of adequate signal amplification for detection by ERG recordings.

As in earlier studies [22], [23], we find that gene-replacement therapy of *rd10* mice using AAV5-PDEβ can significantly postpone light-dependent photoreceptor-cell death, but that degeneration continues over time, albeit at much slower rates than in control retinas. The reason for continued photoreceptor-cell death in retinas treated with AAV5-PDEβ is not known. One possibility is that the AAV5 serotype of the vector used achieves only limited expression of PDEβ, resulting in inadequate control of cGMP levels and increased vulnerability to apoptotic cell death. Consistent with this notion, long-term survival of *rd10* photoreceptor cells has been shown to be improved by introducing PDEβ using a mutant AAV8 (AAV8-733) construct that achieves rapid and high-level expression of the recombinant protein [23].

ERG responses of *rd10* mice are also preserved by AAV5-PDEβ sub-retinal injection, with or without XIAP co-treatment (see below). This protection appears to be greater in animals injected at P21 than P4, but this may simply reflect the way the data were normalized to allow for inter-animal comparison. For each animal, the ERG amplitudes (photopic or scotopic) measured at various times after moving to the light environment were normalized to those of the untreated-fellow eye at time zero (i.e. just before leaving the dark-rearing environment). Thus, at time zero, animals treated at P21 were older and had lower ERG amplitudes than animals treated at P4. When normalized for the reduction in amplitudes, preservation of the ERG signal appears approximately equal in mice treated at either age, consistent with the similar extent of structural preservation in the two groups.

Whatever the limitations of AAV5-PDEβ therapy, we find that a combined anti-apoptotic strategy can significantly enhance the efficacy and duration of therapeutic outcomes. In *rd10* mice injected with both AAV5-PDEβ and AAV5-XIAP, the loss of ONL cells is significantly slower and retinas exhibit higher ERG responses than retinas treated with AAV5-PDEβ alone. In addition, *rd10* mice first treated with AAV5-XIAP at P4, then subsequently treated with AAV5-PDEβ after 2 weeks in the light, respond with a dramatic slowing of further retinal degeneration that is not achieved by gene-replacement therapy in animals of the same age who have not received prior XIAP treatment. However, under the conditions tested so far, this combination approach does not rescue ERG responses, even though the production of proteins involved in visual transduction is increased. This failure appears to be the consequence of the abnormal morphology of photoreceptor outer segments that develops when the animals are placed in the light environment, the underlying causes of which are not known. These structural changes in outer segment structure are not seen when AAV5-PDEβ therapy is administered to dark-reared animals, suggesting a light-dependent aspect to the change in the cells' transcription profile and subsequent degeneration. Despite the absence of ERG responses in the sequentially treated retinas, it is not clear that there is complete absence of visual function. In human studies with RPE65 gene-replacement therapy, there was significant improvement in visual function despite lack of ERG rescue [5]–[7]. Thus, more sensitive techniques for measuring visual function (for example, pupillometry or psychophysical testing) will be needed to determine the full extent of visual function rescue achievable using this approach of photoreceptor preservation with XIAP therapy prior to definitive rescue with gene-replacement therapy.

A general concern with therapies involving injections into the subretinal space is the potential damage caused by the creation of a retinal detachment, which in and of itself can cause outer segment retraction – a phenomenon that reverses when the retina becomes reattached [26]. In the case of sub-retinal injection in the *rd10* mice, the detachment was transient, lasting 24–48 hours, after which AAV5-PDEβ treated retinas exhibited relatively normal appearing outer segments. This suggests that the PDEβ mutation itself, not the creation of the transient detachment, is responsible for photoreceptor and outer segment loss in *rd10* mice, and that AAV5-XIAP therapy improves cell survival but cannot overcome loss of an essential outer segment protein. The mechanism by which the PDEβ mutation affects outer segment formation warrants additional investigation.

In summary, although strategies to block apoptotic cell death have significant limitations as stand-alone therapies for inherited retinal degeneration, they appear to enhance rescue by the corrective gene, as well as permit rescue at older ages than normally possible, thus effectively extending the window-of-opportunity for gene-replacement therapy. Our data suggest that early treatment with AAV5-XIAP could be beneficial, both to extend the timeline for subsequent gene-replacement therapy and to increase the long-term efficacy of such treatment. These benefits need to be weighed against any potential risk of long-term XIAP overexpression in the retina, as XIAP is a powerful inhibitor of programmed cell death that could interfere with normal function or result in transformation events. However, the clear results now obtained support the continued study of anti-apoptotic therapy as an adjunctive treatment, particularly given the slow pace at which gene-replacement vectors are being developed and introduced into clinical practice.
